# Involution of categorical thinking processes in Alzheimer’s disease: Preliminary results

**DOI:** 10.1590/S1980-57642009DN20100012

**Published:** 2008

**Authors:** Claudia Berlim de Mello, Jacqueline Abrisqueta-Gomez, Gilberto Fernando Xavier, Orlando Francisco Amodeo Bueno

**Affiliations:** 1 Department of Psychobiology, Universidade Federal de São Paulo, São Paulo, Brazil. Doutora em Psicologia – USP; pesquisadora do Centro Paulista de Neuropsicologia - AFIP, Associação Fundo de Incentivo à Psicofarmacologia.; 2 Doutora em Ciências, Departamento de Psicobiologia, UNIFESP; Pesquisadora do Centro Paulista de Neuropsicologia - AFIP, Associação Fundo de Incentivo à Psicofarmacologia.; 3 Department of Physiology - USP, Brazil. Professor Adjunto do Instituto de Biociências da Universidade de São Paulo.; 4 Livre Docente em Psicobiologia, Professor Adjunto do Departamento de Psicobiologia - UNIFESP.

**Keywords:** Alzheimer’s disease, neuropsychological tests, memory, thinking, doença de Alzheimer, testes neuropsicológicos, memória, pensamento

## Abstract

Alzheimer’s disease (AD) is a degenerative brain disorder characterized by progressive losses in cognitive functions, including memory. The sequence of these losses may correspond to the inverse order of the normal sequence of ontogenetic cognitive acquisitions, a process named retrogenesis. One of the acquisitions that improve in normal development is the ability to retrieve previously acquired categorical knowledge from semantic memory in order to guide associative thinking and memory processes; consequently, children become able to associate verbal stimuli in more complex taxonomic ways and to use this knowledge to improve their recall.

**Objective:**

In this study, we investigated if AD-related deterioration of semantic memory involves a decrease in categorical thinking processes with progression of the disease, according to the retrogenesis hypothesis.

**Methods:**

We compared the performance of AD patients at mild and moderate stages, and of groups of 7, 10 and 14-year-old children in tasks of free association along with recall tasks of perceptually and semantically related stimuli.

**Results:**

ANOVAS showed a decrease in taxonomic associations and an increase in diffuse associations between mild and moderate stages, corresponding to the inverse order shown by the child groups. At the moderate AD stage, the pattern was similar to that of 7-year-old children. Both groups of patients performed worse than child groups in recall tasks.

**Conclusions:**

These results corroborate the hypothesis of an involution of the processes of categorical associative thinking in the course of the disease.

## Abstract (pt)

A doença de Alzheimer (DA) é uma doença degenerativa do cérebro caracterizada por perdas progressivas nas funções cognitivas, incluindo memória. A seqüência destas perdas pode corresponder à ordem inversa da seqüência normal das aquisições na ontogênese, um processo conhecido como retrogênesis. Uma das aquisições que melhoram no desenvolvimento normal é a habilidade de recuperar conhecimento categórico previamente adquirido da memória semântica de forma a organizar o pensamento associativo e a memória imediata; como conseqüência, as crianças tornam-se capazes de associar estímulos verbais de formas mais complexas, taxonômicas, e de usar este conhecimento para melhorar sua recordação.

**Objetivo:**

Neste estudo, investigamos se a deterioração cognitiva associada à DA envolve uma redução dos processos de pensamento categórico ao longo da progressão da doença, considerando a hipótese da retrogênesis.

**Método:**

Comparamos o desempenho de pacientes com DA nos estágios inicial e moderado e de grupos de crianças com 7, 10 e 14 anos de idade em tarefas de associação livre e recordação de estímulos semântica e perceptivamente associados.

**Resultados:**

ANOVAS mostraram uma redução das associações taxonômicas e um aumento de associações difusas entre os estágios inicial e moderado, correspondendo à ordem inversa mostrada pelos grupos de crianças. No estágio moderado, o padrão foi similar ao das crianças de 7 anos. Os dois grupos de pacientes apresentaram pior desempenho que as crianças nas tarefas de memória.

**Conclusões:**

Os resultados corroboram a hipótese de uma involução dos processos de pensamento associativo categórico no curso da doença.

Reisberg et al.^1,2^ proposed that progression of Alzheimer’s disease (AD) leads to a sequence of cognitive losses corresponding to the inverse order of the normal sequence of ontogenetic cognitive acquisition, a process called retrogenesis. Early symptoms of AD include memory difficulties for recent events. As the disease progresses, AD patients also experience difficulties in accessing general knowledge or facts stored in semantic memory.^3^ Differences in the time course for types of mnemonic deficits seem to occur because some brain structures are more susceptible to neurodegeneration in AD than others. For instance, the hippocampus and the entorhinal cortex, thought to be involved in episodic memory^4^, degenerate earlier compared to frontal, temporal and parietal associative cortex.^5^ Apparently, priority has been given to investigating AD-related episodic memory disruption over semantic memory disruption because this knowledge could help to improve diagnosis in the early phase of the disease.

This study investigated possible losses in categorical associative thinking processes in AD patients at different stages of the disease as a way of evaluating the retrogenesis hypothesis while contributing toward studies on diagnosis at early stages.

## Semantic memory and Alzheimer’s disease

Semantic memory relates to storage of general knowledge about the world, including meaning and concepts.^4^ Concepts seem to be stored as interconnected shared attributes of information, thus forming a complex network,^6^ that include super-ordinate (e.g., the category “animals”) and subordinate (e.g., the item “dog”) relationships. New concepts integrated into this network correspond to the formation of new connections; the stronger these connections are, the easier it is to retrieve this knowledge later. Therefore, establishment of meaningful representations depends upon organization of previously acquired information and incorporation of new knowledge within this integrated network.

The organization of this conceptual network seems to be preserved in healthy elderly people^7-11^ but not in AD patients. For instance, patients in the earlier stages of AD exhibit, relative to healthy matched controls, greater impairment in semantic verbal fluency tasks (e.g., generation of a list of animals) compared to phonological verbal fluency tasks (e.g., generate words starting with a given letter).^12^ Together, these results reveal a semantic memory deficit rather than impairment in accessing information stored in long-term memory.

Evidence from different laboratories suggests that super-ordinate conceptual representations may exhibit greater preservation upon evolution of AD compared to subordinate conceptual representations. For example, Troster et al.^13^ asked moderate AD patients to generate lists of items that can be acquired in supermarkets and found that they generated fewer exemplars per category (e.g., asparagus) and more often referred to super-ordinate category labels (e.g., vegetables) than matched healthy controls. Therefore, the disruption to the structure of semantic memory in AD seems to be marked by an initial loss of subordinate category knowledge and relative preservation of super-ordinate categorical knowledge.

Evidence from free association tasks favors the notion that AD patients exhibit dissociations in their categorical memory. For instance, Hodges, Salmon and Butters^14^ showed that AD patients' ability to associate stimuli according to general categories (e.g., living *versus* non-living things) is greater than their ability to associate specific attributes (e.g., land animals *versus* sea animals). In addition, Chan et al.^15^ showed that AD patients tend to focus on concrete attributes of a given category (e.g., size for animals) instead of semantic attributes (e.g., belonging to the “domestic” category), and Glosser et al.^16^ described that their priming in a word reading test was facilitated when the primer maintains a super-ordinate relationship with the word to be read (e.g., daughter – relative) compared to primers that maintain a coordinate relationship with the word to be read (e.g., cousin – nephew). In contrast, priming effects are subtle or inexistent in both AD patients and matched healthy controls when materials with no preexisting representations in memory are used in testing.^17^

## Development of semantic memory

Semantic network theories postulate that activation of concept-related subcomponents of a network automatically spread to associated concepts;^6^ this spreading activation depends upon the strength of previously established connections. According to this view, it is possible to identify which characteristics receive priority in processing in a given network, allowing evaluation of the knowledge organization within a conceptual domain.

Knowledge organization is related to concept formation, which is developed progressively in childhood. Vygotsky^18^ postulated that while in pre-school concrete thought is the major form of knowledge organization, in early adolescence one develops abstract thought, when association of stimuli depend upon their super-ordinate, taxonomic, relationships. For instance, while pre-school children associate cat and dog relying on their immediate concrete experience, e.g., “because they fight”, adolescents associate them categorically, i.e., “because they are animals”. This latter complex associative thinking is critical for processes of memory acquisition and retrieval of information, being related to the development of memory strategies.^19^

According to the theory of concept formation put forward by Vygotsky,^18^ performance in picture free sorting categorization tasks may be mediated by perceptual, diffuse, functional and taxonomic associations. Perceptual associations rely on specific sensory characteristics of the items to be associated, for instance, their colors. Diffuse association relies on actual or imaginary daily experiences to explain how items are grouped. For instance, to explain the association involving figures of a cat and a bed, the subject might say *“the cat and the bed were put together because the cat sleeps on the bed”*. In other words, the key feature guiding this association relates to a concrete form of thought. Functional associations are established based on conceptual attributes shared by the associated items, including, for instance, the material they are made of (e.g., “bed, bench, table and chair are made of wood”) or some specific function (“one uses glasses and cups to drink from”). Taxonomic associations refer to categories and reveal the occurrence of abstract associative thinking (e.g., “they are animals” or “they are means of transportation”). This latter type of association is considered more elaborate because it requires concepts formation.

In fact, evidence gathered from association of figures tasks suggests that categorization by children depends upon their stage of development; while 7 to 8-year-old children preferably rely on perceptual and diffuse associations, 10 to 12-year-old children rely on functional and taxonomic associations^18,20^.

## The retrogenesis hypothesis and Alzheimer’s disease

According to the retrogenesis hypothesis one might expect AD-related deterioration of semantic memory to involve taxonomic associations in the early stages of the disease, and then encompass functional, diffuse and perceptual associations with progression of the disease. The present study evaluated this hypothesis. Performance of patients at mild and moderate stages of AD was evaluated in a categorical card sorting test, compared to that of groups of children at the ages of 7, 10 and 14 years. If in fact cognitive losses related to conceptual knowledge in AD follow an inverse pattern versus that seen in ontogenetic development, then patients with mild AD symptoms should perform like older children, with prevalence of taxonomic associations in the categorical sorting card test, while patients with moderate AD symptoms should perform similarly to younger children, with prevalence of perceptual and diffuse associations. Furthermore, one should expect these patients to show some preservation of categorical associative thinking, reflecting the influence of semantic memory organization on retrieval processes; if this was the case, one should expect stronger taxonomic associations to be accompanied by better performance on recall tasks.

## Methods

### Participants

This study involved 19 patients with AD (7 men and 12 women), 10 of which were diagnosed as mild stage (3 men and 9 women) and 9 as moderate stage (4 men and 5 women) of the disease. Patients were diagnosed according to the criteria of the National Institute of Neurological Communicative Disorders and Stroke – Alzheimer Disease and Related Disorders Associations.^21^ The level of severity of the patients’ clinical condition was determined by their scores on the Mini-Mental State Exam (MMSE),^22^ validated for Brazilians.^23^ The mean age for the group of patients at the mild stage of the AD was 76.5 years (±6.4) having mean score on the MMSE of 24.1 (±2.6). The mean age for the group of patients at the moderate stage was 75.7 years (±7.2) having a mean score on the MMSE of 19.1 (±2.5). The groups were matched by education level (9 to 11 schooling years). The study also included three groups of children aged seven, ten and fourteen, each group comprising 10 subjects. The children were selected from public schools and all had normal academic performance. The Ethics Committee of our institution approved the research project. The patients’ caregivers and children’s parents signed informed consent forms.

### Procedure

The procedures used to evaluate the subjects followed the guidelines described by Mello^20^. The tests involved thirty white cards measuring 10 x 10 cm, each bearing a picture that was semantically and perceptually related to the other pictures in the set. The figures were obtained from a set of standardized pictures, normalized for name agreement, familiarity and visual complexity, developed by Snodgrass and Vanderwart,^24^ and validated for Brazilians by Pompéia, Miranda and Bueno.^25^ There were five semantically related pictures belonging to six categories (animals, fruits, school material, kitchen utensils, means of transportation and furniture); three ten-picture sets, each of them including items of all semantic categories, were printed using different colors (red, green and yellow).

Subjects were required to perform different tasks using these pictures, including (1) naming, (2) immediate free recall (named “immediate”), (3) free associations (subjects were asked to group the pictures and then to explain the criteria they had used to do so), (4) free recall 20 minutes after re-exposure to the pictures during the free associations task (i.e., the previous task) (named “after re-exposure”); and (5) cued recall (named “cued”). This latter task consisted of oral presentation, by the experimenter, of the label for a specific category for which items had already been recalled by the subject, in order to facilitate semantic processing. For instance, “*you recalled horse and cow, which are animals. Were there other animals in the set of pictures I showed you?*”

Performance of the subjects on each of the recall tests was evaluated by the number of items recalled. Evaluation of performance in the free association task took into account how the subjects explained their associations; as described above, where associations could be classified as (a) perceptual, (b) diffuse, (c) functional, and (d) taxonomic, according to the classification by Vygotsky^18^. The frequency of each type of association was recorded; higher frequencies of taxonomic associations pointed to more abstract associative thinking.

For statistical analysis purposes, the percentage of each type of association in each group was transformed (arcsine √x)^26^ and the resulting values subjected to one-way Analysis of Variance (ANOVA) followed by orthogonal comparisons. Scores achieved by the subjects in the recall tests were compared using ANOVA’s followed by Tukey’s post-hoc tests. Group performances were considered to be statistically different when ANOVA yielded “P-values” less than 0.05.

## Results

### Types of association in the free associations task

**Figure 1:**
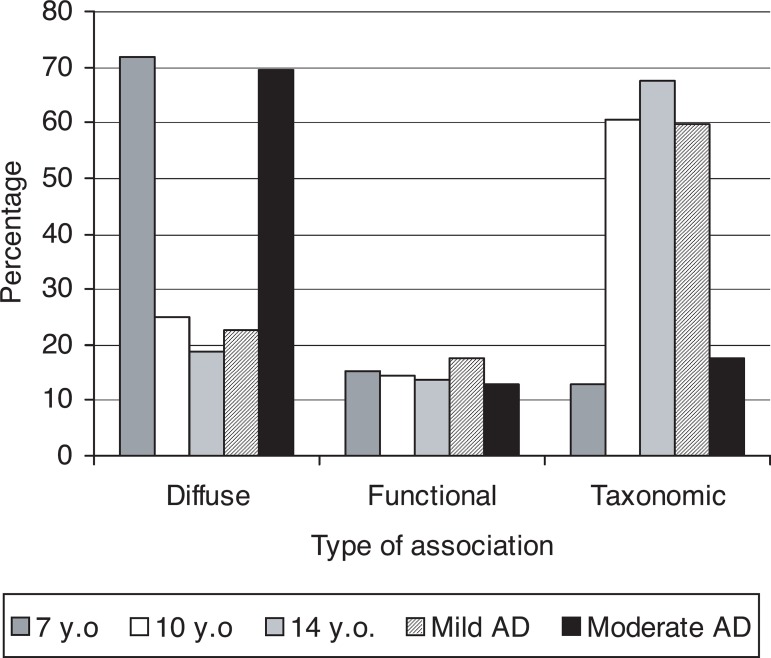
Percentage of each type of figure association in Alzheimer’s disease patients and in child groups.

The percentages of associations classified as “diffuse”, “functional” and “taxonomic” for each of the groups in the free associations task are presented in Figure 1 (note that the “perceptual” category was not presented because no subjects associated stimuli according to color).

Associations of the functional types tended to remain constant (F_4,44_=0.10; p=0.98). Significant differences were observed between groups as regards the percentages of taxonomic (F_4,44_=7.88; p<0.0001) and diffuse (F_4,44_=6.68; p<0.0003) associations. Patients at moderate stages did not differ from 7 year-old children in the percentage of taxonomic (F_1,44_=0,39; p=0.54) and diffuse associations (F_1,44_=0.10; p=0.75). Mild stage patients did not differ from 10 and 14-year old groups in the percentage of diffuse (F_1,44_=0.63; p=0.43) and taxonomic associations (F_1,44_=0.22; p=0.64). But moderate stage patients and 7 year old groups in comparison to mild stage patients and 10 and 14-year old groups differed from each other on percentage of diffuse associations (F_1,44_=25.66; p<0.0001) as well as percentage of taxonomic associations (F_1,44_=30.50; p<0.0001).

These results clearly showed that performance of AD patients at moderate stages of the disease was equivalent to that shown by 7 year-old children in terms of the type of associations performed in this task; that is, both groups relied on diffuse associations 70% of the time in order to perform free associations in the task. On the other hand, performance of patients at mild stages of the AD was equivalent to that seen for 10 and 14 year-old children; i.e., both groups relied on taxonomic associations approximately 60% of the time and on diffuse associations about 25% of the time.

### Free recall tests

**Figure 2:**
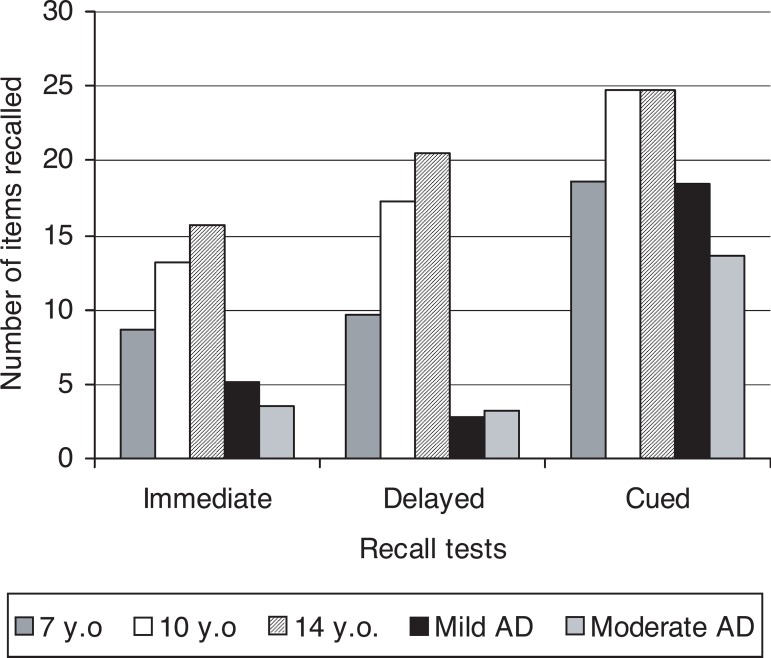
Number of items recalled in the different recall tests by Alzheimer’s disease patients and child groups.

Results also revealed significant differences among the groups on the tests of immediate free recall, recall after re-exposure and cued recall (Figure 2).

The ANOVA showed significant differences between groups in performance on the recall tests (F_4,44_=4.14; p<0.0001). In the groups of children, the number of items recalled increased from one test to the next, and progressively with age. However, the number of items mild AD patients recalled in the immediate recall was lower than the number of items recalled by 10 (p<0.0001) and 14-year old children (p<0.0001). This number did not differ, however, from the number of items obtained by the 7-year-old children (p=0.09). Also, in cued recall, the number of items recalled by mild stage patients was lower than that of 10 (p<0,0001) and 14-year old children (p<0.0001) but again it did not differ from the number obtained by the 7-year-old group (p=1). In delayed recall, however, their performance was also worse than that of the 7-year-old group (p<0.0001). Patients at the moderate stage differed from all the other groups as regards all the recall tests (p<0.0001). No differences were observed between the groups of patients on the immediate (p=0.98) and delayed recall tests (p=1), but in cued recall, those at mild stage recalled a higher number of items (p<0.003). In other words, the groups of patients differed between each other only on cued recall, suggesting that, in initial stages, patients can benefit from the presentation of recall cues more consistently than those at the moderate stage.

## Discussion

The present study aimed at investigating whether there is deterioration in the processes of categorical associative thinking in patients at mild and moderate stages of Alzheimer’s disease. In order to verify this hypothesis, we compared AD-patient performances to those of children of different ages.

The results we obtained in the free association tasks of semantically and perceptually stimuli showed an evolution of these processes in children, as Vygostky^18^ postulated. Between seven and fourteen years of age, there was a progressive substitution of diffuse associations, which express a more concrete associative thinking, by taxonomic associations, which are more abstract in nature. However, the results were different in patients diagnosed with Alzheimer’s disease. Between mild and moderate stages, there was a decrease in taxonomic associations and an increase in the diffuse type. In other words, at the mild stage, the typical pattern of consolidation of concept formation, expressed predominantly by taxonomic associations, remained. At the moderate stage, on the other hand, the pattern was similar to that of 7-year-old children, in whom there is a prevalence of diffuse associations. These results corroborate the hypothesis of an involution of the processes of categorical associative thinking in the course of the disease. These findings therefore confirm the notion of retrogenesis, such as that proposed by Reisberg et al.^2^

Memory skills were also investigated. We observed that the number of items recalled increased progressively from one test to another in the groups of children. This increase can be attributed to the second exposure to the material and also to semantic processing of the stimuli, first provided by free association (between immediate and delayed recall) and subsequently by presentation of verbal labels for the category (between delayed recall and cued recall), when subjects were reminded of previously recalled stimuli. This increase in the number of items recalled was not observed in patients between immediate and delayed recall tests. This finding suggests that memory difficulties are due to impairments in the semantic processing of the information (coding). On the other hand, we observed that the number of items retrieved in cued recall increased, suggesting that the patients are still able to benefit from the presentation of the verbal label of the category. Therefore, the results confirm previous evidence holding that superordinate knowledge is preserved in Alzheimer’s disease.^14,16^ The differences observed between patients at mild and moderate stages, however, indicate that this ability tends to be progressively affected in the course of the disease. It is possible, hence, that the presentation of categorical clues constitutes a feasible resource as a strategy for cognitive rehabilitation of AD patients, in the sense that it might assist them, particularly in early stages, to retrieve information from memory.

In short, the results obtained in the present study support previous evidence suggesting deterioration in the organization of semantic memory in the course of Alzheimer’s disease. In addition, they point to the fact that this deterioration is characterized by an involution to primitive stages, typical of seven-year-old children, and that the superordinate knowledge tends to be preserved. This involution might account for the difficulties patients have in retrieving conceptual information. These must be considered as preliminary results, since they are part of a broader study aimed at analyzing effects of gender and level of education on the processes investigated.
